# Editorial: Parathyroid disorders: updates of PTH/serum Ca2+ regulation and therapeutic prospects

**DOI:** 10.3389/fendo.2023.1354277

**Published:** 2024-01-16

**Authors:** Fan Zhang, Yinde Huang, Jiongyu Hu, Supeng Yin

**Affiliations:** ^1^ Department of Breast and Thyroid Surgery, Chongqing General Hospital, Chongqing, China; ^2^ Department of Endocrinology, Southwest Hospital, Third Military Medical University (Army Medical University), Chongqing, China

**Keywords:** parathyroid, parathyroid hormone, hypoparathyroidism, hyperparathyroidism, GNAS, blastocyst complementation

The parathyroid hormone (PTH) secreted by the parathyroid glands plays a critical role in regulating serum calcium and phosphate levels. Abnormal PTH secretion can lead to a range of diseases, such as hyperparathyroidism and hypoparathyroidism, which can significantly affect bone health, kidney function, and overall metabolic health.

This Research Topic of Frontiers in endocrinology highlights the significant theme of “*Parathyroid Disorders: Updates of PTH/Serum Ca2+ Regulation and Therapeutic Prospects*,” with 11 complete submissions considered, of which five manuscripts have been published. Innovative topics include hypoparathyroidism, primary hyperparathyroidism, PTH resistance, retrospective study, and Mendelian randomization analysis.

Based on the results of retrospective studies, Vincentis et al. reported the importance of the serum Ca/P ratio in the early identification of patients with pseudohypoparathyroidism (PHP) and hypoparathyroidism (HPT). This indicator is particularly valuable for diagnosing patients with atypical phenotypes of parathyroid dysfunction, such as asymptomatic mild hypocalcemia. Additionally, the study found that patients in the pseudohypoparathyroidism type 1B (PHP1B) subgroup exhibited worse biochemical characteristics at diagnosis compared to those in the pseudohypoparathyroidism type 1B (PHP1A) subgroup, such as lower serum Ca and higher serum PTH, resulting in a lower Ca/P ratio. These results fill a gap in the application of PTH and serum calcium in parathyroid diseases.

Molecular biology investigations have helped understand the pathogenesis of PHP. PHP is closely associated with abnormalities in the GNAS gene, with Gsa being the primary transcript of GNAS. Yang et al. summarized the role of the PTH/PTHrP-Gsa-cAMP-PKA signaling pathway in parathyroid diseases. PTH and PTHrP activate Gsa, promoting intracellular cAMP production and initiating the signaling pathway. This pathway leads to a decrease in phosphate levels and an increase in vitamin D levels in the renal tubules, helping maintain normal calcium balance. In autosomal dominant (AD) PHP1B families, A/B hypomethylation is related to differential methylation in the upstream GNAS NESP55 region or maternal microdeletions in the STX16 gene. Recent studies have found that imprinting control regions (ICR), specifically NESP-ICR, is essential for methylation and transcriptional silencing of A/B on the maternal allele, and SXT16-ICR is a long-range enhancer of NESP55 transcription originating from maternal NESP-ICR (1). These results reveal an important GNAS imprinting control mechanism, advancing molecular understanding of PHP1B pathogenesis, and may offer new pathways for PHP treatment.

Primary hyperparathyroidism (PHPT) is often accompanied by other cardiovascular and metabolic diseases. Dobreva et al. analyzed 838 PHPT patients, finding a higher incidence of obesity, type 2 diabetes, arterial hypertension, ischemic heart disease, chronic heart failure, and cerebrovascular atherosclerosis, especially among older patients. Moreover, the study discovered that being over 56 years old, having a glomerular filtration rate below 92 ml/min/1.73m², and a body mass index above 28.3 kg/m² were critical points for increased risk of cardiovascular disease.

Another study indicates an optogenetic approach to regulate PTH secretion, focusing on its potential application in secondary hyperparathyroidism (SHPT). It demonstrates that optogenetic stimulation can elevate intracellular calcium, inhibiting both PTH synthesis and secretion in human parathyroid cells. The study also explores the effects of this approach in a rat model of SHPT, revealing its efficacy in inhibiting *in vivo* PTH release and influencing bone remodeling. Additionally, the study suggests that optogenetic regulation of PTH can be automatically controlled using ionized calcium concentration, offering potential therapeutic applications in managing SHPT and related bone diseases (2).

New clinical analysis methods, such as Mendelian randomization analysis, expand the biological functions of PTH. Jiang et al. explored potential causal relationships between hyperparathyroidism and seven types of blood cell counts and biochemical indicators. The results suggest a negative correlation between hyperparathyroidism and platelet count, and a positive correlation with Mean Platelet Volume and Platelet Distribution Width. These findings provide new evidence of PTH’s influence on platelet activity. Mu et al. investigated factors affecting serum calcium levels and the incidence of hypocalcemia after parathyroidectomy in patients with primary hyperparathyroidism, finding that preoperative serum calcium influences postoperative serum calcium levels, emphasizing the importance of preoperative internal environment grouping for individualized treatment. Annebäck et al. reported that 8.7% of 1636 Swedish patients who underwent total thyroidectomy developed permanent hypoparathyroidism. Among them, nearly a quarter had PTH levels below the normal range within 24 hours post-surgery, and later, this condition developed into permanent hypoparathyroidism in 6.7% of cases. These findings underscore the importance of monitoring PTH levels within the first 24 hours after surgery for the early detection and treatment of hypoparathyroidism (3). PTH replacement therapy is significant for hypoparathyroidism. The PaTHway, a 26-week phase III clinical trial, assessed the efficacy and safety of TransCon PTH (palopegteriparatide) in treating hypoparathyroidism. 84 participants were randomized to receive daily TransCon PTH (starting dose 18μg/d) or placebo, both with standard care. The primary efficacy endpoint was participants achieving normal serum calcium levels at week 26 without standard care and no increase in medication dose in the 4 weeks prior to week 26. The results showed significant improvements with TransCon PTH in all key secondary efficacy endpoints compared to placebo, with most adverse events being mild or moderate. In summary, TransCon PTH maintained normal calcium levels in patients with hypoparathyroidism and was well tolerated. A systematic review found that PTH treatment reduced the use of vitamin D and calcium supplements and increased the incidence of hypercalcemia, recommending PTH treatment for long-term management of hypoparathyroidism (4). Considering the high cost and potential complications of recombinant human PTH treatment, a recent study used blastocyst complementation (BC) technology to produce functional parathyroid gland (PTG) cells as a new method for treating permanent hypoparathyroidism. Successful generation of parathyroid-deficient embryos for BC was achieved by knocking out the Gcm2 gene using CRISPR-Cas9. In these embryos, mouse embryonic stem cells (mESCs) differentiated into mature PTGs, rescuing the neonatal death of Gcm2 knockout mice. These mESC-derived PTGs responded to extracellular calcium, restoring calcium homeostasis in mice with surgically induced hypoparathyroidism. The study also successfully generated functional interspecies PTGs in newborn rats with Gcm2 gene knockout, offering potential for future heterologous animal BC treatments for human PTG diseases (5). The results suggest that BC technology can produce functional endocrine organs, and combined with organoid technology, it provides a new perspective for treating hypoparathyroidism.

## Conclusion

This Research Topic enhances our understanding of serum calcium and PTH in parathyroid diseases, highlighting the significance of clinical characteristics in diagnosing these disorders. It underscores the need for clinicians to improve both preoperative and postoperative management of thyroid and parathyroid conditions. These insights offer fresh perspectives on the development and treatment of parathyroid diseases.

## Author contributions

FZ: Writing – original draft. YH: Writing – original draft. JH: Writing – review & editing. SY: Writing – review & editing.
